# Effects of Organic and Conventional Growing Systems on the Phenolic Profile of Extra-Virgin Olive Oil

**DOI:** 10.3390/molecules24101986

**Published:** 2019-05-23

**Authors:** Anallely López-Yerena, Julián Lozano-Castellón, Alexandra Olmo-Cunillera, Anna Tresserra-Rimbau, Paola Quifer-Rada, Brígida Jiménez, Maria Pérez, Anna Vallverdú-Queralt

**Affiliations:** 1 Department of Nutrition, Food Science and Gastronomy XaRTA, Institute of Nutrition and Food Safety (INSA-UB), Faculty of Pharmacy and Food Sciences, University of Barcelona, 08028 Barcelona, Spain; naye.yerena@gmail.com (A.L.-Y.); jullozcas@gmail.com (J.L.-C.); alexandra.oc.94@gmail.com (A.O.-C.); mariaperez@ub.edu (M.P.); 2 CIBER Physiopathology of Obesity and Nutrition (CIBEROBN), Institute of Health Carlos III, 28029 Madrid, Spain; anna.tresserra@iispv.cat; 3 Human Nutrition Unit, University Hospital of Sant Joan de Reus, Department of Biochemistry and Biotechnology, Faculty of Medicine and Health Sciences, Pere Virgili Health Research Center, Universitat Rovira i Virgili, 43002 Reus, Spain; 4 Department of Endocrinology & Nutrition, CIBER of Diabetes and Associated Metabolic Diseases (CIBERDEM), Biomedical Research Institute Sant Pau, Hospital de la Santa Creu i Sant Pau, 08041 Barcelona, Spain; pquifer@santpau.cat; 5 Instituto Andaluz de Investigación y Formación Agraria, Pesquera, Alimentaria y de la Producción Ecológica, Centro de Cabra, Antigua Ctra, Cabra-Doña Mencía, Km. 2.5, 14940 Córdoba, Spain; brigida.jimenez@juntadeandalucia.es; 6 Laboratory of Organic Chemistry, Faculty of Pharmacy and Food Sciences, University of Barcelona, 08028 Barcelona, Spain

**Keywords:** phenolic compounds, Hojiblanca, variety, organic, conventional, agriculture, mass spectrometry, oleocanthal, secoiridoids, ripening, NMR

## Abstract

Extra-virgin olive oil (EVOO) is largely appreciated for its proven nutritional properties. Additionally, organic foods are perceived as healthier by consumers. In this context, the aim of the present study was to compare the phenolic profiles of EVOO from olives of the Hojiblanca variety, cultivated under organic and conventional systems. The quantification and identification of individual polyphenols was carried out by liquid chromatography coupled to mass spectrometry in tandem mode (LC-MS/MS). Significantly higher levels (*p* < 0.05) of phenolic compounds were found in organic EVOOs. The methodology used was able to detect previously unreported differences in bioactive components between organic and conventional EVOOs.

## 1. Introduction

Extra virgin olive oil (EVOO), a key component of the Mediterranean diet, is highly appreciated for its nutritional and organoleptic attributes. The minor compounds include aliphatic and triterpene alcohols, sterols, hydrocarbons, volatile compounds and antioxidants such as carotenoids and polyphenols, which contribute to the organoleptic characteristics, stability and nutritional value of EVOO [1,2]. The qualitative and quantitative composition of polyphenols in EVOO is affected by many variables, such as the degree of olive ripeness, the technological production process and storage conditions [3,4]. The most important changes in the polyphenol content occur during the crushing and malaxation of olives [5,6] as well as during the storage and filtration of EVOO [7]. Other influential factors include the use of organic and conventional growing systems [8,9].

The market for organic products, generally perceived as healthier and safer than conventional foods, is growing annually [10], despite the higher costs and lower productivity of organic compared to traditional agriculture. In 2016, on a global level, up to 178 countries practiced organic agriculture, on an extension of 57.8 million hectares, with a market size of 89.7 billion US dollars [11]. A key difference between the two growing systems is soil fertility management, which can affect the nutritive composition of plants, including levels of secondary metabolites [12]. In organic agriculture, which is associated with the promotion of biodiversity and biological cycles, crops obtain nitrogen and nutrients from a diverse soil ecosystem. Contrastingly, conventional farming uses fertilizers containing soluble inorganic nitrogen and other nutrients, which are more directly available to plants [13]. Phenolic biosynthesis in plants is known to be strongly affected by the cultivar, the environmental conditions (especially light), as well as the type of fertilization [14,15]. Previous studies demonstrate that the organic fruits have higher phenolic content than conventional ones [16,17,18].

Therefore, the objective of our study was to compare the content of polyphenols (secoiridoids, flavones, phenolic alcohols, phenolic acids and lignans) in Hojiblanca EVOO produced by organic and conventional production systems under the same environmental conditions. Moreover, we applied a quantitative ^1^H nuclear magnetic resonance (qNMR) method to corroborate the concentration of oleocanthal (OLC) in our EVOOs obtained by LC-MS.

## 2. Results

### 2.1. Total Amount of Phenolic Compounds

**Figure 1:**
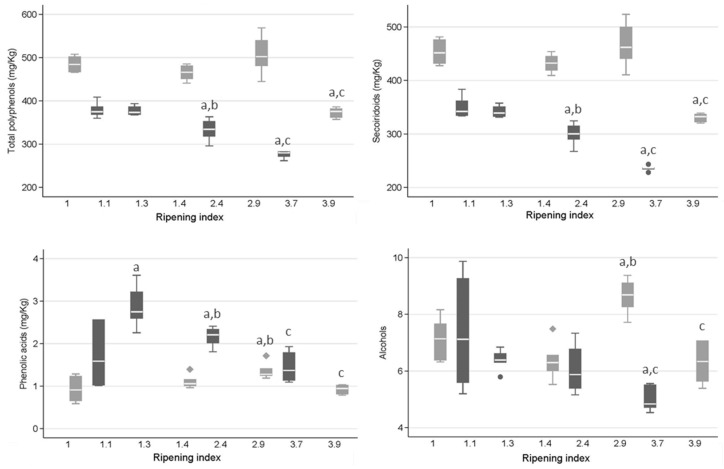

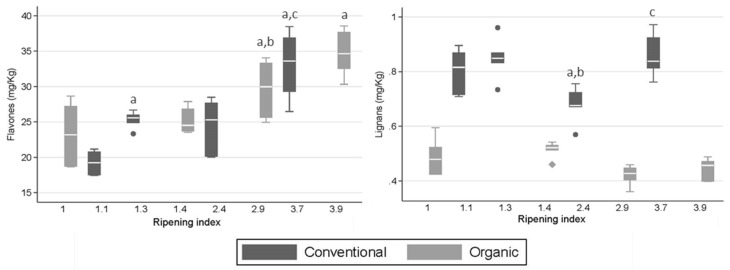
Boxplots for concentrations (mg·kg^−1^) of total phenols (TP) and polyphenolic groups in extra-virgin olive oil (EVOO) by ripening index (RI) and growing method. (a means *p* < 0.05 vs. 1st RI, b means *p* < 0.05 vs. 2nd RI, c means *p* < 0,05 vs. 3rd RI within the same growing.

The average of total phenols (TP) in organic and conventional EVOO, with the *p*-value for the differences assessed by the Mann–Whitney test, are shown in Table 1. In order to control the ripening factor when assessing the differences in phenolic concentrations between the two types of EVOO, generalized linear models adjusting for ripening index (RI) were used (Table S1). The mean TP content of organic and conventional EVOO was 456.89 ± 56.74 and 338.19 ± 42.96 mg·kg^−1^, respectively, being 26% higher in EVOO produced by the organic system (Figure 1).

### 2.2. Concentrations of Phenolic Groups and Selected Phenolic Compounds

The major phenolic compounds in EVOO were secoiridoids (SEC), whereas lignans, phenolic acids and flavones were present in low concentrations. The SEC represented 91–92% of the phenolic compounds, with higher levels in the organic than conventional EVOO (420.72 ± 59.42 and 306.48 ± 48.09 mg·kg^−1^, respectively). OLC was the predominant ligstroside derivative found in organic and conventional EVOO samples (186.72 ± 40.61 and 132.10 ± 37.02 mg·kg^−1^, respectively), being 30% higher in the former. Also, the elenolic acid concentration was positively affected by the organic system (55.35 ± 8.10 mg·kg^−1^), being 27% lower in conventional EVOO (40.37 ± 7.39 mg·kg^−1^).

Luteolin was the predominant flavonoid in both organic and conventional EVOO (22.69 ± 5.09 and 19.35 ± 5.38 mg·kg^−1^, respectively) and the apigenin represented 20–24% of the total flavonoids and its content was not affected by the agronomic conditions (6.17 ± 0.78 and 5.51 ± 0.69 mg·kg^−1^, conventional and organic, respectively).

The concentration of the total phenolic alcohols was not affected by the organic or conventional growing systems (7.11 ± 1.15 and 6.21 ± 1.37 mg·kg^−1^, respectively). However, the content of hydroxytyrosol was higher under the organic than the conventional system (4.47 ± 1.10 and 3.65 ± 1.32 mg·kg^−1^, respectively).

The content of lignans and phenolic acids, which are important phenolic components of EVOO, were higher under the conventional system (0.79 ± 0.09 and 2.05 ± 0.71 mg·kg^−1^, respectively). The only lignan found was pinoresinol and the phenolic acids were *p*-coumaric, ferulic and vanillic acid (1.04 ± 0.36, 0.07 ± 0.01, and 0.93 ± 0.37 mg·kg^−1^, respectively).

### 2.3. EVOO Phenolic Profile and Olive Fruit Ripening

The concentration of total SEC, phenolic acids, flavones and lignans in EVOO samples extracted from olives of the Hojiblanca cultivar, grown in conventional and organic conditions and harvested at different RI, are presented in Figure 1. To assess the effect of the RI of the olives on the content of phenolic compounds in conventional and organic EVOO, regression models were fitted (Table 2).

The concentration of TP and SEC decreased during ripening in both conventional and organic EVOO (Table 2). Conversely, an increase in the content of flavones was correlated with ripeness in both organic and conventional EVOO (*p* < 0.001). The total phenolic acids and phenolic alcohols were affected by the olive ripening stage only in the conventional system, showing lower levels with later harvests. Lignans were not affected by the RI in any system.

### 2.4. Analysis of Oleocanthal by NMR

The qNMR showed that the level of OLC was higher (168.96 mg·kg^−1^) in the EVOO made from organic vs. conventionally cultivated olives (118.21 mg·kg^−1^) (Table 3). Thus, the significant variation in OLC concentrations among the samples of the Hojiblanca EVOO is in accordance with previous studies recently reviewed [19] and the results of our OLC analysis by mass spectrometry (MS).

**Figure 2:**
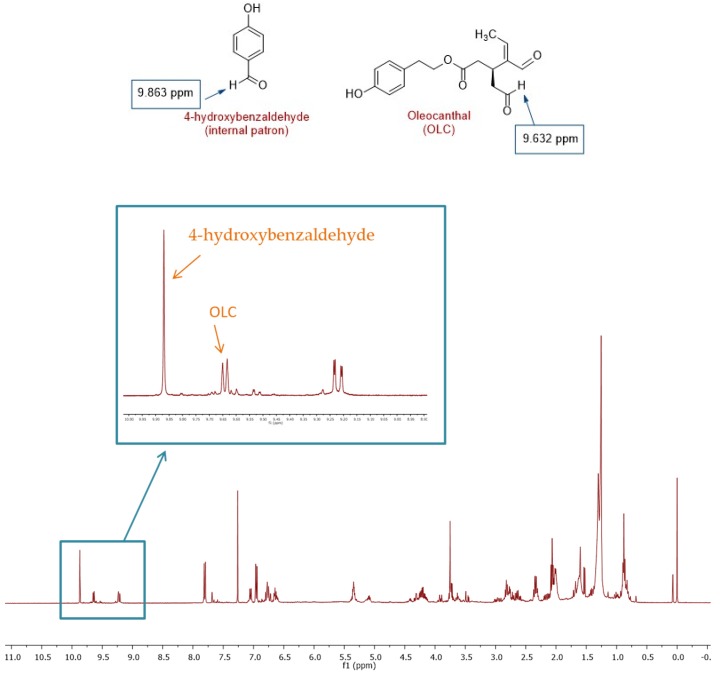
^1^H NMR spectrum of EVOO acetonitrile extracts recorded in CDCl_3_ and using 4-hydroxybenzaldehyde as the internal standard. The signals of the OLC and internal standard are shown in the expanded spectrum.

As 1D ^1^H NMR typically provides an excellent linear response to component concentrations, it was envisaged as a simple and reliable methodology to validate the UPLC-MS monitoring of OLC levels. The aldehydic proton region of the target compound in the ^1^H NMR spectrum of EVOO acetonitrile extracts, when recorded in CDCl_3_, presented a well-resolved set of peaks, making feasible the integration of one of the aldehydic protons and its comparison with the peak of the internal standard. OLC was quantified by integrating the singlet at 9.632 ppm (Figure 2).

## 3. Discussion

### 3.1. Total Amount of Phenolic Compounds

Recent studies found a higher polyphenol content in organic EVOO [20] and a different acid composition, as well as a higher degree of bitterness (cv. Leccino and Frantoio) and pungency (cv. Frantoio) and less sweetness (cv. Frantoio) [21]. A similar enhancing effect of organic cultivation on TP content was observed in EVOO extracted from olives of the Casaliva variety, both unripe (51% increase) and ripe (40% increases), whereas in a multi-varietal organic EVOO this effect was only observed with unripe olives [22]. However, in other studies, agronomic factors did not play a clear role in the TP content of EVOO made from olives of different cultivars, which instead was mainly affected by the year of the harvest [8], or water availability [23]. The type of farming (organic or conventional) only becomes a major factor in the TP content of tomatoes [24], pepper [25] and fruits [26] when these are grown under similar environmental conditions.

### 3.2. Concentrations of Phenolic Groups and Selected Phenolic Compounds

Organic agriculture is associated with a natural increase in the amount of defense substances, as the plant is exposed to greater stress in the absence of synthetic pesticides. In addition, without synthetic fertilizers there is less bioavailable nitrogen, with concomitant lower plant growth rates and an enhanced production of secondary metabolites such as phenolic compounds [27,28].

Previous studies have established that SEC (oleuropein and oleuropein, ligstroside and elenolic acid derivatives) are the most complex and abundant family of polyphenols in EVOO polar fractions [29,30,31] and are the principal contributors to organoleptic traits [32]. SEC are synthesized through the secologanin pathway, which does not depend on nitrogen or phosphorus, so their production is not impeded with the low nitrogen and phosphorus availability of organic soil [33]. A recent study demonstrated that the foliar fertilization with a biofertilizer rich in calcium increased oleuropein aglycone and OLC levels in EVOO, which in contrast, decreased significantly with the use of a biofertilizer rich in nitrogen, phosphorus and potassium [34]. Therefore, conventional practices could explain the lower content of most of the SEC compounds (oleuropein derivatives and ligstrosides derivatives) compared to organic practices. The ester breakdown of SEC leads to the formation of elenolic acid and derivatives [35]. In our study, the elenolic acid concentration was in accordance with previously reported results in different EVOO (16.8–58.6 mg·kg^−1^) [36,37]. Since the content of SEC was higher in organic EVOO, it is also logical to have higher content of elenolic acid (Table 1), because the more SEC, the more elenolic acid is released from SEC breakdown.

The main flavonoids present in EVOO are luteolin and apigenin [38,39] and a high luteolin content is of great interest, as it has been associated with health-promoting and antioxidant properties of foods [40,41]. The luteolin concentration reported here (Table 1) is higher than in other studies (3.12 and 7.57 mg·kg^−1^) [36,42], including one in which EVOO was also extracted from Hojiblanca olives (3.69–6.67 mg·kg^−1^) [43]. These differences among studies are not surprising, since it has been shown that the concentration of luteolin depends considerably on the olive variety, geographical area, season, environmental conditions and cultivation method [36,44]. In another study, the use of a biofertilizer rich in nitrogen, phosphorus and potassium led to a significant increase in the apigenin content of EVOO (1.66 ± 0.32 mg·kg^−1^), whereas luteolin levels increased when the biofertilizer was supplemented with calcium (2.12 ± 0.39 mg·kg^−1^) [34].

In our work, the concentration of the total phenolic alcohols is similar to that of another study comparing organic and conventional EVOO made from the same type of olives (8.33–11.0 and 10.5–16.3 mg·kg^−1^, respectively) [43]. Other authors reported a decrease in hydroxytyrosol concentration in EVOO when olive trees were fertilized with nitrogen, phosphorus and potassium, as occurred with SEC [34]. The phenolic alcohols are derived from the SEC, so their biosynthesis does not depend on the nitrogen or the phosphorus [33].

Both lignans and phenolic acids are synthesized through the phenylpropanoid pathway [45,46] and depend on the shikimic pathway, in which nitrogen and phosphorus take part [47]. The greater availability of nitrogen and phosphorus in conventional farming could thus explain the lower lignan and phenolic acid concentration in the organic EVOO.

### 3.3. EVOO Phenolic Profile and Olive Fruit Ripening

During ripening, the chemical structure and concentrations of compounds in olives can be modified by chemical reactions and the enzymatic activity of glycosidases, phenol oxidases and phenol polymerases [48,49]. The amount of these enzymes depends on the cultivar and maturation stage [41]. Thus, the degree of olive fruit ripeness is a crucial parameter in EVOO quality [50]. Previous studies have reported a reduction in TP, beginning at a maturation index of 2.5–3 [51], or a significant gradual decrease from the first to the fifth harvest [23]. It has been suggested that the TP content depends more on the olive cultivar than an early or late harvest [52].

The amount of SEC decreases significantly with ripeness, both in organic and conventional systems, as reported in the literature [48,53,54]. The SEC concentration was found to decrease by 31% (92.1–63.0 mg·kg^−1^) between the first and last harvests (maturation index of 2.4–5.6), due to oleuropein degradation during ripening [55]. Also, Gutierrez-Rosales et al. [56] showed that high contents of oleuropein aglycone at the initial stage of ripening were caused by a high activity of *β*-glucosidase. This indicates that oleuropein biosynthesis combines with enzymatic hydrolysis to produce the aglycone form. Thus, when the olive is in a green stage, the level of *β*-glucosidase activity increases proportionally with the amount of oleuropein and ligstroside, whereas in the black stage, when the phenolic glycoside concentration is reduced, the glucosidase activity is low [49].

Reports in the literature on the influence of ripening on flavonoid content are contradictory. The content of flavones (luteolin and apigenin) in olives was observed to increase up to a maturation index close to 4, decreasing thereafter [38], whereas elsewhere this tendency was found at an index of 0.76–1.27 [54]. Furthermore, an increase in flavonoid concentration has been reported in EVOO made from olives at an intermediate ripening stage [57,58]. In Hojiblanca EVOO, a higher content of luteolin was obtained by harvesting medium-ripe olives (6.10 and 6.59 mg·kg^−1^, conventional and organic EVOO, respectively), whereas an early harvest resulted in increased apigenin (3.32 and 3.65 mg·kg^−1^, conventional and organic EVOO, respectively) [43]. The association of a higher concentration of luteolin with an intermediate ripening stage could be because apigenin is the substrate for a hydroxylase enzyme in the flavonoid pathway, giving rise to luteolin [53].

With respect to phenolic acids, our results are in agreement with those of Jimenez et al., who reported that *p*-coumaric and ferulic acid contents were higher in EVOO extracted from olives at an early RI, decreasing progressively thereafter, and that the concentration of vanillic acid was apparently not affected by the ripening process [59]. Another study found an increase in phenolic acids in EVOO extracted from more mature olives, which may be due to the activity of hydrolytic enzymes on the complex phenols [53].

### 3.4. Analysis of Oleocanthal by NMR

The various extraction procedures and analytical methods developed for the quantification of EVOO phenolic compounds have generated ambiguous results that are difficult to compare. The most commonly used methods are liquid chromatography (LC), followed by UV-Vis or detection by MS [60,61]. However, OLC can react with different solvents and consequently both liquid–liquid extraction and chromatographic analysis may interfere with its determination by LC-MS, leading in some cases to broader or multiple peaks in MS detection. [62]. A promising new method has been published recently by Sánchez de Medina et al. [63], but further studies are needed to assess its accuracy and reliability. For this reason, the chromatographic results obtained with this method were validated by acetonitrile extraction from random samples of EVOO and directly measured OLC levels by qNMR, as described by Karkoula et al. [64]. The interest of qNMR is due to the repeatability and reproducibility of measurements, as well as its rapidity compared to more classical methods, and its reliability [65]. Other advantages include simple sample preparation, low sample consumption and non-destructive measurement.

This validation study was performed by NMR using CDCl_3_ as the solvent, avoiding both the undesired interactions of other solvents like methanol or water with the target compound, and the overlap of the aldehydic proton peaks. We chose 4-hydroxybenzaldehyde as the internal standard due to its price, stability, and solubility in our deuterated solvent, and the simplicity of the resulting ^1^H NMR spectrum. Moreover, the aldehydic proton peaks of this compound and OLC do not overlap.

## 4. Material and Methods

### 4.1. Chemicals

OLC was purchased from PhytoLab GmbH (Vestenbergsgreuth, Germany); oleuropein, lutein, *m*-coumaric acid, pinoresinol, lariciresinol, isolariciresinol, secoisolariciresinol and taxifolin were obtained from Sigma-Aldrich (Madrid, Spain). *p*-Coumaric acid, vanillic acid, ferulic acid and apigenin were obtained from Fluka (Buchs, Switzerland), hydroxytyrosol from Extrasynthese (Genay, France), and verbascoside from HWI ANALYTIK GmbH (Rülzheim, Germany). Hexane, methanol, acetonitrile, and chloroform-d were purchased from Sigma-Aldrich and cyclohexane from Carlo Erba (Madrid, Spain).

### 4.2. Olive Fruit Samples

Olive fruits were collected from olive trees of the Hojiblanca cultivar, which were cultivated using organic and conventional agricultural practices without irrigation. The orchard was located on the experimental farm of the Agricultural Research Training Centre in Cabra in the province of Cordoba, at an altitude of approximately 547 m. The soil pH was 8 and its composition was limestone and sand. The climate was continental Mediterranean, with hot summers and cold winters, and the average temperature between October of 2017 and January of 2018 was 12.7 °C, with an average relative humidity of 64.4%. Ten trees were selected per cultivation system. The olives were harvested on 4 different days with 2 weeks of difference between every picking.

The RI of each harvest was determined according to the methodology proposed by Uceda and Frías [66], which is based on the color of the skin and the pulp. 100 olives were randomly selected and the following formula was applied: RI = (A × 0 + B × 1 + C × 2 + D × 3 + E × 4 + F × 5 + G × 6 + H × 7) /100. Where A, B, C, D, E, F, G, H are the number of olives with the 8 different ripening stages. Those are: stage 0: intense green skin; stage 1: yellowish green skin; stage 2: green skin with red spots, in less than half of the fruit; stage 3: reddish or purple skin in more than half of the fruit; stage 4: black skin and white pulp; stage 5: black skin and pulp purple; stage 6: black skin and more than half of the pulp purple; stage 7: black skin and totally purple pulp. The fruit RI in organic and conventional system was 1 to 3.945 and 1.06 to 3.68., respectively.

### 4.3. Oil Samples

Three representative olive samples, each weighing a minimum of one kilogram, were processed and the corresponding EVOOs were obtained using an Abencor milling system (Abengoa S.A., Seville, Spain). This system reproduced the industrial process on a laboratory scale. The apparatus consisted of three elements: a hammer mill, a thermobeater and a pulp centrifuge. The olive fruits (6 kg) were milled using a stainless-steel hammer mill equipped with a 5-mm sieve that was operated at 3000 rpm. The resulting olive paste was immediately kneaded in a mixer at 50 rpm for 30 min at 30 °C, with hot water added at 20 min. Centrifugation of the kneaded olive paste was performed in a basket centrifuge at 3500 rpm for 1 min. After centrifugation, the oil was decanted and stored in amber glass bottles at 4 °C in darkness and without headspace until analysis.

### 4.4. Polyphenol Analysis by Liquid Chromatography

The liquid–liquid extraction of phenolic compounds was performed with the method proposed by Capriotti et al. [67]. 1 g of EVOO was dissolved in hexane (oil/hexane 1:1, *w*/*v*) in a 10 mL centrifuge tube and shaken for 30 s. The polyphenols were extracted with 2 mL of MeOH and stirred for 30 s; the emulsion was then centrifuged at 3000 rpm and 4 °C for 3 min. The supernatant (methanolic extract) was subjected to a second cleaning with hexane, and the hexane extract was subjected to a second extraction of polyphenols with MeOH. All extracts were shaken for 30 s and centrifuged at 3000 rpm and 4 °C for 3 min. The methanolic extracts were recovered and cleaned up by dispersing 50 mg of C18. The samples were evaporated and reconstituted with 800 μL of MeOH:H_2_O (80:20 *v*/*v*), filtered with (Polytetrafluoroethylene) PTFE syringe filters (0.2 µm), transferred to an amber glass vial and stored at −80 °C until analysis. The internal standard was added to the EVOO to obtain a final concentration of 5 ppm after the reconstitution. The experiment was done in triplicate.

The identification and quantification of phenolic compounds was performed using an Acquity^TM^ UPLC (Waters; Milford, MA, EUA) coupled to an API 3000 triple-quadruple mass spectrometer (PE Sciex) with a turbo ion spray source. Separation of compounds was achieved using an Acquity UPLC^®^ BEH C_18_ Column (2.1 × 50 mm, i.d., 1.7 µm particle size) and Acquity UPLC^®^ BEH C_18_ Pre-Column (2.1 × 5 mm, i.d., 1.7 µm particle size) (Waters Corporation^®^, Ireland) (See supporting information). The mobile phases were H_2_O with 0.2% acetic acid (A) and ACN (B). An increasing linear gradient (*v*/*v*) of B was used (t (min), %B), as follows: (0, 5); (2.5, 5); (12.5, 40); (12.6, 100); (13.5, 100); (13.6,5); (15,5), at a constant flow rate of 0.4 mL/min. The injection volume was 10 µL and the column temperature 40 °C.

The quantification of OLC was performed using a methodology proposed by Sánchez de Medina et al. with some modifications. Separation was achieved using an Acquity UPLC^®^ BEH C_18_ Column (2.1 × 50 mm, i.d., 1.7 µm particle size) and Acquity UPLC^®^ BEH C_18_ Pre-Column (2.1 × 5 mm, i.d., 1.7 µm particle size) (Waters Corporation^®^, Ireland). The mobile phases were MeOH (A) and H_2_O (B), both with 0.1% of formic acid. An increasing linear gradient (*v*/*v*) of B was used (t (min), %B), as follows: (0, 100); (2, 100); (4.75, 46.4); (4.9, 0); (5.9, 0); (6.100); (6.5, 100), at a constant flow rate of 0.6 mL·min^−1^. The injection volume was 5 µL and the column temperature 50 °C. The MS potentials were optimized for the compound (Supporting Table S2). Method suitability was evaluated by submitting random samples to a comparative NMR study.

Ionization was achieved using an electrospray interface operating in the negative mode [M–H] and all the compounds were monitored in the multiple monitoring mode (MRM) with the following settings: capillary voltage, −3500 V; nebuliser gas (N_2_), 10 (arbitrary units); curtain gas (N_2_), 12 (arbitrary units); and drying gas (N_2_) heated to 450 °C. The declustering potential, focusing potential, collision energy and entrance potential were optimized to detect phenolic compounds with the highest signals, following the method described by Suárez et al. [39]. The system was controlled by Analyst version 1.4.2 software supplied by Applied Biosystems.

The calibration curves were prepared in refined oil and were linear over the concentration ranges 0–20 mg·mL^−1^ using oleuropein, hydroxytyrosol, *p*-coumaric acid, *m*-coumaric acid, vanillic acid, ferulic acid, apigenin, luteolin, pinoresinol, lariciresinol, isolariciresinol, secoisolariciresinol, verbascoside and OLC.

### 4.5. Analysis of Oleocanthal by NMR

The OLC extraction and sample preparation for NMR analysis were carried out using the methodology proposed by Karkoula et al. [30]. Olive oil (8.0 g) was mixed with cyclohexane (32 mL) and ACN (40 mL). The mixture was homogenized using a vortex mixer for 30 s and centrifuged at 4000 rpm for 5 min. The ACN phase (40 mL) was collected, mixed with 1.6 mL of 4-hydroxybenzaldehyde solution (0.5 mg·mL^−1^) in ACN, and evaporated under reduced pressure using a rotary evaporator (Buchi, Model R-200 with dry ice and acetone cold-trap condenser, Switzerland).

The residue of the above procedure was dissolved in CDCl_3_ (750 μL), and an accurately measured volume of the solution (550 μL) was transferred to a 5 mm NMR tube. ^1^H NMR spectra were recorded at 400 MHz using an NMR spectrometer (Varian VNMRS 400 MHz). Typically, 128 scans were collected into 32K data points over a spectral width of 16 ppm (6410 Hz), with a relaxation delay of 1 s and an acquisition time of 2.5 s. The spectra were phase corrected and integrated automatically using MNova. Accurate integration was performed manually for the peaks of interest.

### 4.6. Statistical Analysis

Significant differences between organic and conventional samples were assessed by the Mann–Whitney test (Table 1). The relationship between categorical exposure variables (organic vs conventional cultivation) and concentration of polyphenols was assessed by Generalized Linear Models adjusting for RI (Table 2). Regression models were also fitted to assess associations between polyphenol concentration as a dependent variable and RI as an independent variable. Statistical analyses were conducted using STATA software (version 14.0; StataCorp, College Station, TX, USA). *p* values < 0.05 were considered statistically significant.

## 5. Conclusions

The TP in EVOO made from Hojiblanca olives were analyzed, comparing organic and conventional growing systems under the same environmental conditions, and levels were significantly higher in organic samples (*p* < 0.05). The concentration of SEC, which are synthesized through the secologanin pathway without the need for nitrogen or phosphorus, was higher in oils from olives cultivated under organic conditions. These included oleocanthal, which was satisfactorily analyzed by LC-MS/MS, as demonstrated by results obtained by qNMR.

In contrast, the concentrations of lignans and phenolic acids were higher under the conventional system, as their synthesis is through the phenylpropanoid pathway via shikimic acid, which requires nitrogen and phosphorus. In the case of phenolic alcohols and flavones, there were no significant differences associated with the cultivation method.

When the effect of the ripening stage of the olive fruit was assessed, both the TP and SEC concentrations were found to decrease with maturation in both production systems, whereas the flavone content increased. Olive maturation was also associated with a decline in certain compounds: lignans in organic EVOO and phenolic acids and phenolic alcohols in conventional samples.

It should be emphasized that long-term experiments are required to eliminate the effect of seasonality. There is also a need for more randomized, controlled dietary intervention trials to corroborate the potentially greater beneficial effects of organic food on human health compared to those produced conventionally. However, organic food may be recommended, not only for its health benefits, but also because its production has less of an environmental impact.

## Figures and Tables

**Table 1:** Phenolic compound contents (mg·kg^−1^) of conventional and organic EVOO made from Hojiblanca olives. * *p*-values of U Mann–Whitney test.

	Organic	Conventional	*p* *
**Total Phenols**	456.89 ± 56.74	338.19 ± 42.96	<0.001
**Secoiridoids**	420.72 ± 59.42	306.48 ± 48.09	<0.001
Oleuropein	0.82 ± 0.02	0.81 ± 0.02	0.2
Oleuropein derivatives			
Oleuropein der I	22.77 ± 3.01	34.83 ± 4.44	<0.001
Oleuropein der II	3.21 ± 0.54	1.67 ± 0.25	<0.001
Oleuropein der III	3.63 ± 0.58	2.33 ± 0.33	<0.001
me-3,4-DHPEA-EA	1.46 ± 0.25	0.97 ± 0.07	<0.001
Hydroxy oleuropein aglycone I (HOA I)	1.44 ± 0.34	1.17 ± 0.18	0.007
Hydroxy oleuropein aglycone II (HOA II)	2.18 ± 0.77	1.67 ± 0.41	0.02
HDCM OA	9.13 ± 3.53	6.37 ± 1.94	0.01
3,4-DHPEA-EA I	7.18 ± 0.98	4.74 ± 0.42	<0.001
3,4-DHPEA-EA II	5.82 ± 0.94	3.02 ± 0.74	<0.001
Lactone	0.19 ± 0.04	0.33 ± 0.20	<0.001
Ligstroside derivatives			
Ligstroside I	20.45 ± 2.77	12.32 ± 2.47	<0.001
Ligstroside II	41.61 ± 3.68	24.41 ± 4.02	<0.001
Ligstroside III	54.59 ± 10.63	34.75 ± 8.00	<0.001
Oleocanthal	186.72 ± 40.61	132.10 ± 37.02	<0.001
Elenolic acid	55.35 ± 8.10	40.37 ± 7.39	<0.001
Elenolic acid derivatives			
Hydroxyelenolic acid	3.41 ± 1.42	3.19 ± 1.70	0.5
**Flavones**	28.21 ± 5.55	25.53 ± 5.85	0.09
Luteolin	22.69 ± 5.09	19.35 ± 5.38	0.03
Apigenin	5.51 ± 0.69	6.17 ± 0.78	0.008
**Phenolic alcohols**	7.11 ± 1.15	6.21 ± 1.37	0.07
Hydroxytyrosol	4.47 ± 1.10	3.65 ± 1.32	0.01
Dihydroxytyrosol	1.73 ± 0.09	1.78 ± 0.10	0.001
3,4-DHPEA-AC	0.91 ± 0.02	0.91 ± 0.03	0.51
**Lignans**	0.47 ± 0.06	0.79 ± 0.09	<0.001
Pinoresinol	0.47 ± 0.06	0.79 ± 0.09	<0.001
**Phenolic acids**	1.08 ± 0.26	2.05 ± 0.71	<0.001
Ferulic acid	0.05 ± 0.01	0.07 ± 0.01	0.003
*p*-coumaric acid	0.67 ± 0.15	1.04 ± 0.36	<0.001
Vanillic acid	0.35 ± 0.12	0.93 ± 0.37	<0.001

**Table 2:** Linear regressions (polyphenol content vs. RI) by type of cultivation.

	Organic		Conventional	
	Coefficient	*p*	Coefficient	*p*
Total phenols	−27.4	0.004	−40.2	<0.001
Secoiridoids	−31.2	0.001	−44.7	<0.001
Phenolic alcohols	0.05	0.81	−0.76	0.002
Phenolic acids	0.02	0.74	−0.29	0.04
Flavones	3.77	<0.001	4.23	<0.001
Lignans	−0.02	0.01	0.003	0.9

**Table 3:** Concentration of oleocanthal (OLC) measured by qNMR in EVOOs of Hojiblanca variety.

	Integration	Concentration (mg·kg^−1^)
Conventional	0.477	118.21
Organic	0.684	168.96

## Supplementary Materials

The following are available online, Table S1: GLM model adjusted for ripeness (conventional vs. organic); Table S2: Multiple reaction Monitoring conditions for the polyphenols.
