# Supplementary material for: Effects of Organic and Conventional Growing Systems on the Phenolic Profile of Extra-Virgin Olive Oil
Source: Molecules. 2019 May 23;24(10):1986. doi: 10.3390/molecules24101986 (PMC6572524; doi:10.3390/molecules24101986)
Supplement: Supplementary file 1 [file molecules-24-01986-s001.pdf]

	Coef. (95% CI)	P
**Total phenols**	121.9 (101.3 to 142.5)	<0.001
**Secoiridoids**	120.2 (100.2 To 140.3)	<0.001
Oleuropein	0.008 (-0.004 To 0.02)	0.19
Oleuropein derivatives		
Oleuropein der I	-12.2 (-14.3 To -10.2)	<0.001
Oleuropein der II	1.50 (1.34 To 1.66)	<0.001
Oleuropein der III	1.24 (1.06 To 1.42)	<0.001
me-3,4-DHPEA-EA	0.48 (0.40 To 0.56)	<0.001
Hydroxy oleuropein aglycone I (HOA I)	0.30 (0.16 To 0.42)	<0.001
Hydroxy oleuropein aglycone II (HOA II)	0.58 (0.30 To 0.85)	<0.001
HDCM OA	2.95 (1.48 To 4.43)	<0.001
3,4-DHPEA-EA I	2.41 (2.00 To 2.81)	<0.001
3,4-DHPEA-EA II	2.69 (2.41 To 2.97)	<0.001
Lactone	-0.14 (-0.21 To -0.06)	<0.001
Ligstroside derivatives		
Ligstroside I	7.38 (7.19 To 9.57)	<0.001
Ligstroside II	17.5 (15.7 To 19.4)	<0.001
Ligstroside III	21.0 (17.6 To 24.4)	<0.001
Oleocanthal	58.8 (42.9 To 74.8)	<0.001
Elenolic acid	15.3 (11.0 To 19.6)	<0.001
Elenolic acid derivatives		
Hydroxyelenolic acid	0.40 (-0.2 To 1.01)	0.2
**Flavones**	1.98 (-0.05 To 4.01)	0.06
Luteolin	2.67 (0.94 To 4.40)	0.003
Apigenin	-0.69 (-1.10 To -0.28)	0.001
**Phenolic alcohols**	0.96 (0.26 To 1.66)	0.007
Hydroxytyrosol	-0.01 (-0.02 To 0.01)	0.41
Dihydroxytyrosol	0.11 (0.05 To 0.16)	<0.001
3,4-DHPEA-AC	0.86 (0.18 To 1.53)	0.01
**Lignans**	-0.33 (-0.37 To -0.28)	<0.001
Pinoresinol	-0.33 (-0.37 To -0.28)	<0.001
**Phenolic acids**	-0.95 (-1.25 To -0.65)	<0.001
Ferulic acid	-0.01 (-0.02 To -0.01)	<0.001
p-coumaric acid	-0.37 (-0.53 To -0.21)	<0.001
Vanillic acid	-0.57 (-0.72 To -0.42)	<0.001

Table s 1. GLM model adjusted for ripeness (conventional vs. organic).

Phenolic compounds	m/z	DP	FP	EP	CE	RT
Oleuropein	539/275	-30	-140	-10	-30	7.68
Oleuropein der I	377/241	-30	-140	-10	-30	9.14
Oleuropein der II	377/307	-30	-140	-10	-30	8.64
Oleuropein der III	377/307	-30	-140	-10	-30	10.32
me-3,4-DHPEA-EA	391/255	-30	-140	-10	-30	11.2
Hydroxy oleuropein aglycone I (HOA I)	393/257	-30	-140	-10	-30	7.6
Hydroxy oleuropein aglycone II (HOA II)	393/257	-30	-140	-10	-30	8.8
HDCM OA	335/199	-30	-140	-10	-30	7.32
3,4-DHPEA-EA I	377/307	-30	-140	-10	-30	10
3,4-DHPEA-EA II	377/307	-30	-140	-10	-30	7.91
Lactone	321/185	-40	-250	-10	-20	7.16
Ligstroside I	361/291	-30	-140	-10	-30	9.01
Ligstroside II	361/291	-30	-140	-10	-30	10
Ligstroside III	361/291	-30	-140	-10	-30	11.2
Oleocanthal	303/285	-40	-170	-5	-10	5.92
Elenolic acid	241/127	-30	-140	-10	-30	6.31
Hydroxyelenolic acid	257/137	-30	-140	-10	-30	6.02
Luteolin	285/133	-100	-340	-10	-50	8.15
Apigenin	269/117	-70	-200	-10	-50	9.11
Hydroxytyrosol	153/123	-40	-250	-10	-20	1.13
Dihydroxytyrosol	169/125	-40	-250	-10	-20	1.09
3,4-DHPEA-AC	195/180	-30	-140	-10	-30	5.69
Pinoresinol	357/151	-60	-180	-8	-25	8.58
Ferulic acid	193/134	-40	-170	-10	-20	5.15
p-coumaric acid	163/119	-40	-150	-10	-25	4.27
Vanillic acid	167/123	-30	-200	-10	-30	2.56

DP: declustering potential; FP: focusing potential; EP: entrance potential; CE: collision energy; RT: retention time

Table s 2. Multiple reaction Monitoring conditions for the polyphenols
